# Levels and determinants of child wasting relapse: a prospective cohort study from Somalia

**DOI:** 10.7189/jogh-16-04019

**Published:** 2026-02-13

**Authors:** Kemish Kenneth Alier, Shelley Walton, Samantha Grounds, Sydney Garretson, Said Aden Mohamoud, Mohamud Ali Nur, Sadiq Mohamed Abdiqadir, Mohamed Billow Mahat, Michael Ocircan P’Rajom, Meftuh Omer Ismail, Abdullahi Abdulle Farah, Qundeel Khattak, Lilly Schofield, Marina Tripaldi, Fabrizio Loddo, Pierluigi Sinibaldi, Farhan Mohamed, Abdifatah Ahmed Mohamed, Adam Abdulkadir Mohamed, Nadia Akseer

**Affiliations:** 1 Johns Hopkins University, Department of International Health, Baltimore, USA; 2 Save the Children, Somalia Country Office, Mogadishu, Somalia; 3 Save the Children, Save the Children International, London, UK; 4 Federal Government of Somalia, Ministry of Health & Human Service, Mogadishu, Somalia

## Abstract

**Background:**

Understanding the rates and determinants of severe acute malnutrition (SAM) relapse is crucial for stakeholders in Somalia, where evidence is limited. This study aimed to assess SAM relapse rates and associated risk factors among children discharged from outpatient therapeutic programmes (OTP) in the Bay and Hiran regions of Somalia.

**Methods:**

We conducted a prospective cohort study of 160 children aged 7–53 months discharged as recovered from OTP SAM treatment between August–September 2023. Children were followed monthly for six time points post-discharge. Anthropometric measurements, morbidity data, and household information were collected. Survival analysis was used to calculate cumulative incidence of SAM relapse, defined by weight-for-height z-score (WHZ)<−3 standard deviation (SD) or mid-upper arm circumference (MUAC)<11.5 cm or oedema. Cox proportional hazard models identified factors associated with relapse.

**Results:**

Cumulative incidence of SAM relapse at Time 1 (T1) = 5.2% (95% confidence interval (CI) = 2.5, 10.6%), T2 = 14.3% (95% CI = 9.4, 21.5%) and T6 = 26.0% (95% CI = 19.3, 34.5%) by WHZ and 13.2% (95% CI = 8.8, 19.5%) by MUAC. The relapse rate for combined SAM and moderate acute malnutrition by WHZ at T1 = 26.9% (95% CI = 19.5, 36.3%), T2 = 36.2% (95% CI = 28.0, 46.1%) and T6 = 50.1% (95% CI = 41.0, 60.0%). Weight-for-height z-score (WHZ)-based relapse was higher in rural areas (31.4% *vs*. 22.7% urban, *P* = 0.285) and among children with WHZ<−3 SD at admission (37.4% *vs*. 21.2%, *P* = 0.029). Mid-upper arm circumference (MUAC)-based relapse was higher in urban areas (20.8% *vs*. 4.1% rural, *P* = 0.002), among younger children (19.7% *vs*. 5.5% > 2 years, *P* = 0.009), and internally displaced persons (21.8% *vs*. 5.8% non-internally displaced persons, *P* = 0.003). Factors significantly associated with increased relapse risk included WHZ<−3 SD at admission (adjusted hazard ratio (HR) = 2.22; 95% CI = 1.04, 4.72) and longer OTP stay (adjusted HR = 1.02 per day; 95% CI = 1.00, 1.04). Participation in a cash assistance programme was protective (adjusted HR = 0.44; 95% CI = 0.22, 0.90).

**Conclusions:**

Severe acute malnutrition (SAM) relapse rates in Somalia are considerable, with varying patterns by anthropometric indicator, region, and demographic factors. Cash assistance programme offers a promising complementary intervention. These findings can inform targeted interventions and policy changes to reduce relapse and improve long-term outcomes for children recovering from SAM in Somalia and similar contexts.

**Registration:**

The cluster-RCT associated with this cohort study is registered at ClinicalTrials.gov, ID: NCT06642012.

Acute malnutrition, also known as wasting, is a major global health concern, impacting an estimated 45 million children under five (CU5) worldwide [1]. While progress has been made against other indicators of malnutrition, such as stunting, the prevalence of wasting has not seen a similar decline and is a significant contributor to CU5 morbidity and mortality [2]. In high-burden settings, children are screened for wasting by community health workers, family members and health workers and referred to Integrated Management of Acute Malnutrition (IMAM) programmes for treatment [3]. Once a child achieves recovery criteria (weight-for-height z-score (WHZ) ≥ −2 and/or mid-upper arm circumference (MUAC) ≥ 125 mm and no oedema for at least two weeks), they are discharged from the programme [4]. While most children treated for severe acute malnutrition (SAM) successfully recover, they can experience higher nutritional vulnerability post-treatment than their never-wasted counterparts. In a matched cohort study of CU5 in Ethiopia, post-SAM children experienced a greater burden of illness and relapse than healthy peers in the same village [5]. Recent systematic reviews show that child wasting relapse rates vary widely from 0–37% globally, with follow-up times ranging from one week to 18 months, and relapse risk highest within the first 3–6 months [6]. Post-discharge mortality is also highest among post-SAM children [7].

Somalia, an impoverished country of 18 million people, struggles with conflict, environmental disasters, extreme food insecurity and high rates of malnutrition [8]. This study addresses critical evidence gaps in Somalia's wasting response. While a recent multi-country study reported 5% SAM relapse using MUAC-only criteria in urban Mogadishu [9], our study uniquely examines both WHZ and MUAC-based relapse across rural and urban populations in two high-burden regions during a different seasonal period. Unlike previous single-site studies, we provide the first comparative analysis of relapse determinants across diverse livelihood zones (pastoral, agropastoral, internally displaced person (IDP)) and evaluate the protective effect of cash assistance programmes in this context [9,10].

While frameworks exist for understanding drivers of wasting relapse, and some empirical work has been done, global relapse rates vary widely by definition: WHZ-based studies report 15–37% (Ethiopia: 25%, Niger: 28%) while MUAC-only studies show 5–15% (Mali: 12%, Democratic Republic of Congo: 8%). These methodological differences, combined with varying follow-up periods (1–18 months), underscore the need for standardised approaches to enable evidence-based programming.

In settings such as Nepal, Niger, Ethiopia, Mali, and Nigeria, evidence from Somalia is sparse [4,11–15]. Since risk and protective factors vary notably by context, understanding factors affecting relapse in Somalia is crucial for effective policy and programming. This study seeks to address this evidence gap by estimating wasting relapse rates, particularly SAM relapse rate and determinants among CU5 discharged from outpatient therapeutic feeding programmes in two high-burden settings of Somalia, Bay and Hiran. Evidence from this study could inform funding, policy and programming efforts for identifying and treating children at high risk of relapse in Somalia and similar humanitarian settings.

## METHODS

### Study setting

During our study, Somalia ranked first on the Fragile States Index [16] with more than half of the 18 million population living in poverty [17]. Food insecurity is highly prevalent with up to one in five Somalis classified by Integrated Food Security Phase Classification as facing severe hunger in 2024 and over 1.7 million CU5 were acutely malnourished [18]. This study was conducted in the Bay and Hiran regions of southwestern Somalia. These two regions were selected due to the high prevalence of acute malnutrition and food insecurity. Additionally, a Bureau for Humanitarian Assistance (BHA) programme (supported by United States Agency for International Development) which focused on providing life-saving cash for nutrition to communities, was being implemented in these regions.

**Figure 1:**
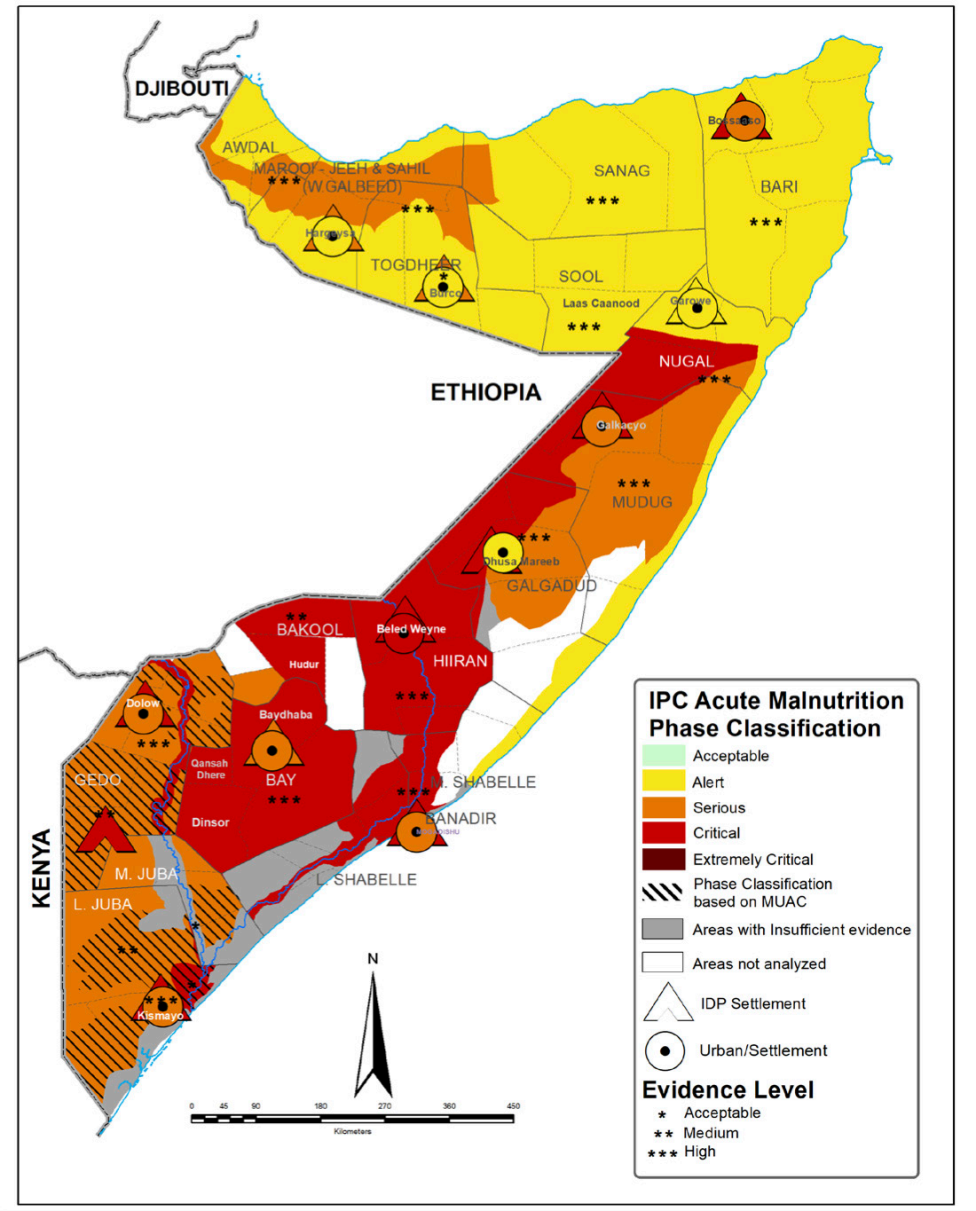
Map of Somalia showing burden of acute wasting by region (October 2023–February 2024) [18]. The figure is free to use under Creative Commons Attribution-Noncommercial-Share-Alike 4.0 International Deed (CC BY-NC-SA 4.0) (https://creativecommons.org/licenses/by-nc-sa/4.0/).

The Bay region has a semi-arid climate and is prone to recurrent droughts and floods, which negatively impact agricultural production and food security. The region has a predominantly agropastoral economy, with many households relying on livestock rearing and crop cultivation as their primary livelihood activities [19]. The Hiran region experiences a semi-arid climate and is also vulnerable to climatic shocks, such as droughts and floods. The region's economy is primarily based on pastoralism, with many households heavily dependent on livestock for their livelihoods [20]. Both the Bay and Hiran regions have been affected by years of conflict, insecurity, food crises, and environmental degradation, which have contributed to high levels of acute malnutrition among CU5 [21] and as represented in **Figure 1** of acute malnutrition between October 2023–February 2024 [18].

### Study design

This was a prospective cohort study for SAM children discharged from OTP and followed up for relapse. The study was conducted in Somalia between August 2023–April 2024 in health facilities supported by Save the Children and follows the Somali guidelines for integrated management and treatment of acute malnutrition [22]. The OTP details are summarised in Table S4 in the **Online Supplementary Document****.**

We employed a convenience sampling approach enrolling children who had been treated and discharged from an OTP programme in the study regions. As this was a descriptive study, we aimed to reach a maximum sample size from all eligible children, as resources and time allowed.

**Figure 2:**
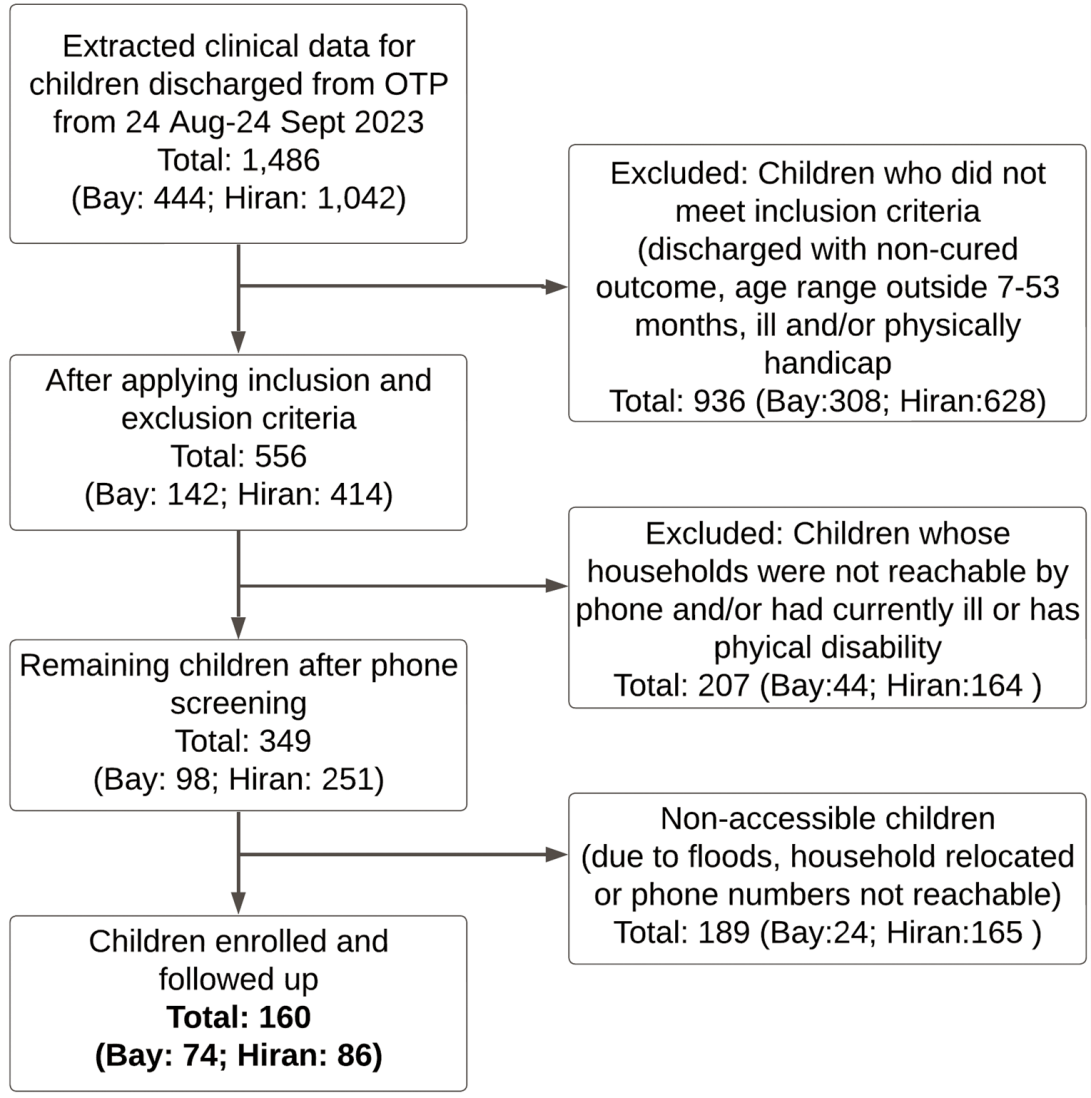
Study recruitment and follow-up flowchart.

We extracted the admission and discharge demographic and clinical data from registers of 22 health facilities (15 Hiran, seven Bay) for children discharged from OTP between 24 August and 19 September 2023, and screened them for enrolment (**Figure 2**). Inclusion criteria included children having no medical complications, being aged 7–53 months at OTP exit, and being discharged with a recovered outcome based on Somalia IMAM guidelines (MUAC ≥ 11.5 cm and/or WHZ ≥ −3 standard deviation (SD)) and no oedema [22]. We excluded children with presence of a chronic or congenital disease (not including HIV infection or tuberculosis) or disability that affects growth, ability to consume food, or anthropometric measurements (*e.g.* cerebral palsy), or if the initial OTP admission was preceded by enrolment in an inpatient, hospital, or stabilisation centre where the child was being treated for SAM with medical complications similar to previous studies [9,23].

Subsequently, we phoned caregivers of the eligible children to determine additional inclusion information as follows:

1) presence of the household in the two study regions and if they intend on staying in the same location for the next six months

2) to ascertain if the child has any chronic medical condition not captured in the medical register

3) if they were willing to participate in the study.

The final study enrolled a total of 160 children (Bay = 74, Hiran = 86) who met all inclusion criteria and whose families provided consent to participate. Consent was confirmed at each follow up point.

### Data collection

Enrolled post-SAM children were followed up monthly at home for six follow-ups post-discharge to assess for SAM as well as linear growth, morbidity, and mortality outcomes. We aimed to follow up children soon after discharge (baseline). The average time for first follow-up post discharge was 60 ± 15 days (hereafter Time 1(T1)). The second to the sixth follow-up were 30 days (one month) apart on average (hereafter Time 2 (T2), Time 3 (T3), Time 4 (T4), Time 5 (T5) and Time 6 (T6)). The first follow-up was conducted from 18–26 November 2023 and the final follow-up was conducted from 15–20 April 2024 corresponding to the second raining season (deyr) and dry season (Jilaal). We use standard anthropometric measurement methods [24].

At the first follow-up, we collected detailed individual, household, and community-level information:

1) individual: anthropometry, age, gender, illness in past two weeks, infant and young child feeding, vaccine status, minimum dietary diversity for children, wasting treatment programme experience

2) household: maternal weight, household size/composition, decision making, assets, food consumption score, hygiene practices, mother education, status of household head, type of water source, household food security indictors (food consumption score, household hunger scale and reduced coping strategies index)

3) community: IDP status, recent shocks experience (floods) and whether household was receiving cash transfers from the BHA-supported project.

The questionnaire is in Table S1 in the **Online Supplementary Document**. At subsequent follow-ups, only child anthropometry and morbidity data were collected. Data quality was ensured through standardised training, inter-observer reliability checks (± 0.5 cm MUAC, ± 0.1 kg weight), and weekly supervision visits. All measurements were double-entered with WHO-standard extreme value flagging. Covariate selection was guided by the United Nations International Children’s Emergency Fund conceptual framework of malnutrition, examining immediate (child factors), underlying (household factors), and basic determinants (displacement, shocks, social protection).

### Study outcomes

The primary outcome of interest was cumulative incidence of SAM defined by WHZ or MUAC or oedema. We defined SAM relapse as children whose WHZ score falls below −3 SD during follow-up or MUAC measurement below 11.5 cm and no oedema. Our secondary outcome was cumulative incidence of moderate acute malnutrition (MAM) for a sub-set of post-SAM children. We defined MAM relapse as any child whose WHZ score falls below −2 SD. We didn’t use MUAC as there were no children with MUAC > 12.5 cm at enrolment. We defined relapse according to Somalia's national IMAM guidelines, consistent with WHO 2006 growth standards. Primary outcome (SAM relapse) was defined as re-diagnosis of SAM (WHZ < −3 SD or MUAC < 11.5 cm or presence of oedema) in children previously discharged as recovered from SAM treatment. Secondary outcome examined relapse to any acute malnutrition (combined SAM + MAM) defined as WHZ < −2 SD among children who achieved complete recovery (WHZ ≥ −2 SD) at discharge.

We used the zscore06 Stata module (StataCorp, College Station, Texas, USA) based on the 2006 WHO child growth standards to calculate anthropometric indices (WHZ, weight-for-age z-score (WAZ), height-for-age z-score (HAZ)) [25,26]. Extreme values were flagged and excluded according to WHO recommendations: WHZ < −5 or > 5, WAZ < −6 or > 5, and HAZ < −6 or > 6.

We constructed three analysis cohorts based on the MUAC and WHZ score at OTP exit:

1) cohort for WHZ ≥ −3 SD at exit and followed up for relapse to WHZ < −3 SD (SAM)

2) cohort for MUAC ≥ 11.5 cm at exit and followed up for relapse to MUAC < 11.5 cm (SAM)

3) cohort for WHZ ≥ −2 SD at exit and followed up for relapse to WHZ < −2 SD (Both SAM + MAM).

We created these cohorts because the heath facilities discharge criteria varied. While some use only MUAC, others used WHZ for discharging children from OTP. The criteria for discharge didn’t always align with criteria used for admission. The cohorts allowed us to examine the different exit scenarios.

### Statistical analysis

Descriptive statistics were calculated for baseline characteristics, including demographics, anthropometry, and household factors. Continuous variables were summarised as means and SDs, and categorical variables were presented as frequencies and percentages. We used *t* tests for continuous variables and χ^2^ tests for categorical variables to assess differences between regions. For each cohort, we conducted time-to-event analyses using Kaplan-Meier survival curves to estimate the cumulative incidence of relapse at six different time points (T1–T6) post-OTP exit. The log-rank test was used to compare survival curves across regions, age groups, urban/rural, and severity of wasting at admission. We calculated incidence rates of relapse with 95% confidence intervals (CIs).

To identify factors associated with relapse, we performed bivariate and multivariable Cox proportional hazards regression analyses. Variables with *P* < 0.2 in bivariate analyses were included in the initial multivariate model. We used a backward stepwise approach to build the final model, retaining variables with *P* < 0.05. The proportional hazards assumption was tested using Schoenfeld residuals. We reported unadjusted and adjusted hazard ratios (HR) with 95% CIs. The final model was adjusted for child age, sex, livelihood zone, and time to relapse. To account for potential clustering effects at the OTP site level, we used robust standard errors in our regression models. We conducted sensitivity analyses to assess the impact of different definitions of relapse using combinations of MUAC and WHZ and to evaluate the effect of children we were not able to reach after extraction of clinic data. All statistical tests were two-sided, and *P*-values < 0.05 were considered statistically significant. We followed STROBE guidelines for reporting observational studies [27]. Data were analysed using Stata version 18 (StataCorp, College Station, Texas, USA).

## RESULTS

A total of 160 post-SAM children were successfully recruited and followed for the duration of the study. There was no dropout during the follow-up period.

### Child and household characteristics

Post-SAM children were predominantly female and aged two years and below (**Table 1**). Complete follow-up was achieved for all 160 participants (100% retention rate) with no censoring events, eliminating potential bias from missing data.

**Table 1:** Characteristics of children and household at baseline*

		By Region		
	**Overall**	**Bay**	**Hiran**	***P*-value**
**Sample**	160	74 (46.2)	86 (53.8)	
**Sex**				
Male	72 (45.0)	37 (50.0)	35 (40.7)	0.238
Female	88 (55.0)	37 (50.0)	51 (59.3)	
**Age, months**				
<2 y	82 (51.2)	56 (75.7)	26 (30.2)	<0.001
≥2 y	78 (48.8)	18 (24.3)	60 (69.8)	
**Household characteristics**				
Rural/urban				
*Rural*	73 (45.6)	0 (0.0)	73 (84.9)	<0.001
*Urban*	87 (54.4)	74 (100.0)	13 (15.1)	
Livelihood zone				
*Pastoral*	45 (28.1)	0 (0.0)	45 (52.3)	<0.001
*Agropastoral*	41 (25.6)	0 (0.0)	41 (47.7)	
*IDP*	74 (46.2)	74 (100.0)	0 (0.0)	
Save the Children BHA Programme†				
*Not in BHA Programme*	56 (35.0)	33 (44.6)	23 (26.7)	0.018
*In BHA Programme*	104 (65.0)	41 (55.4)	63 (73.3)	
Shocks (affected by floods during study)				
*Not flood affected*	38 (30.4)	12 (16.4)	26 (50.0)	<0.001
*Flood affected*	87 (69.6)	61 (83.6)	26 (50.0)	
Admission profile				
*WHZ score (mean/SD)*	−2.5 (1.3)	−2.1 (1.1)	−2.8 (1.4)	<0.001
*WHZ≥−3 to<−2*	44 (28.0)	23 (31.1)	21 (25.3)	
*WHZ<−3 SD*	63 (40.1)	15 (20.3)	48 (57.8)	
*MUAC, cm, mean (SD)*	11.4 (0.7)	11.1 (0.7)	11.6 (0.6)	<0.001
Wasting, MUAC				
*≥11.5 to <12.5*	30 (18.8)	3 (4.1)	27 (31.4)	
*<11.5*	121 (75.6)	71 (95.9)	50 (58.1)	
Oedema at admission				
*Yes*	6 (3.8)	0 (0.0)	6 (7.0)	0.021
Discharge/enrolment profile, mean (SD)				
*Length of stay, days*	42.4 (18.3)	34.2 (10.9)	49.5 (20.4)	<0.001
*WHZ score*	−1.4 (1.4)	−1.0 (1.1)	−1.7 (1.6)	<0.001
*MUAC, cm*	12.0 (0.6)	11.9 (0.2)	12.1 (0.7)	0.002
Underweight, WAZ				
*WAZ≥−3 to<−2*	57 (35.6)	35 (47.3)	22 (25.6)	0.001
*WAZ<−3 SD*	31 (19.4)	17 (23.0)	14 (16.3)	
Stunting, HAZ				
*HAZ≥−2 SD*	72 (45.9)	17 (23.0)	55 (66.3)	<0.001
*HAZ<−2 SD*	85 (54.1)	57 (77.0)	28 (33.7)	
IMAM programme quality				
*Child was not transferred from OTP to TSFP*	79 (49.4)	27 (36.5)	52 (60.5)	0.002
*Child was transferred from OTP to TSFP*	81 (50.6)	47 (63.5)	34 (39.5)	

BHA – Bureau for Humanitarian Assistance, HAZ – height-for-age z-score, IDP – internally displaced person, IMAM – Integrated Management of Acute Malnutrition, MUAC – mid-upper arm circumference, OTP – outpatient therapeutic feeding programme, SBCC – social and behaviour change communication, SD – standard deviation, TSFP – Targeted supplementary feeding programme, WAZ – weight-for-age z-score, WHZ – weight-for-height z-score

*Presented as n (%) unless specified otherwise.

†There was an ongoing BHA livelihood programme implemented by Save the Children in the two regions for vulnerable households. The BHA interventions included multi-purpose cash transfers and SBCC interventions.

Notably, Bay region had a significantly higher proportion of younger children (81.1%) compared to Hiran (31.4%, *P* < 0.001). Household characteristics differed between regions. Bay was exclusively urban (100%) and comprised entirely of IDPs, while Hiran was predominantly rural (84.9%) with a mix of pastoral (52.3%) and agropastoral (47.7%) livelihoods (*P* < 0.001). More households were affected by the floods shock in Bay (83.6%) than Hiran (50.0%, *P* < 0.001).

At admission, the overall mean WHZ was −2.5 ± 1.3 SD, with Hiran showing more severe wasting (WHZ < −3, 57.8%) than Bay (20.3%, *P* < 0.001). Mean MUAC at admission was 11.4 ± 0.7 cm, and Bay (11.1 ± 0.7 cm) had a lower mean MUAC than Hiran (11.6 ± 0.6 cm, *P* < 0.001). The overall length of stay in OTP SAM treatment was 42.4 ± 18.3 days, with children staying longer in Hiran (49.5 ± 20.4 days) than Bay (34.2 ± 10.9 days, *P* < 0.001). Discharge WHZ was −1.4 ± 1.4 SD overall, with Bay (−1.0 ± 1.1 SD) having greater mean WHZ than Hiran (−1.7 ± 1.6 SD, *P* < 0.001). Mean MUAC at discharge was 12.0 ± 0.6 cm, with slight regional variation (Bay = 11.9 ± 0.2 cm, Hiran = 12.1 ± 0.7 cm, *P* = 0.002). Over half of the children were stunted (HAZ<−2 SD) at discharge. Almost half of the children (49.3%) were not transferred to Targeted Supplementary Feeding Programme (TSFP) treatment after exiting OTP.

### Relapse incidence

**Figure 3:**
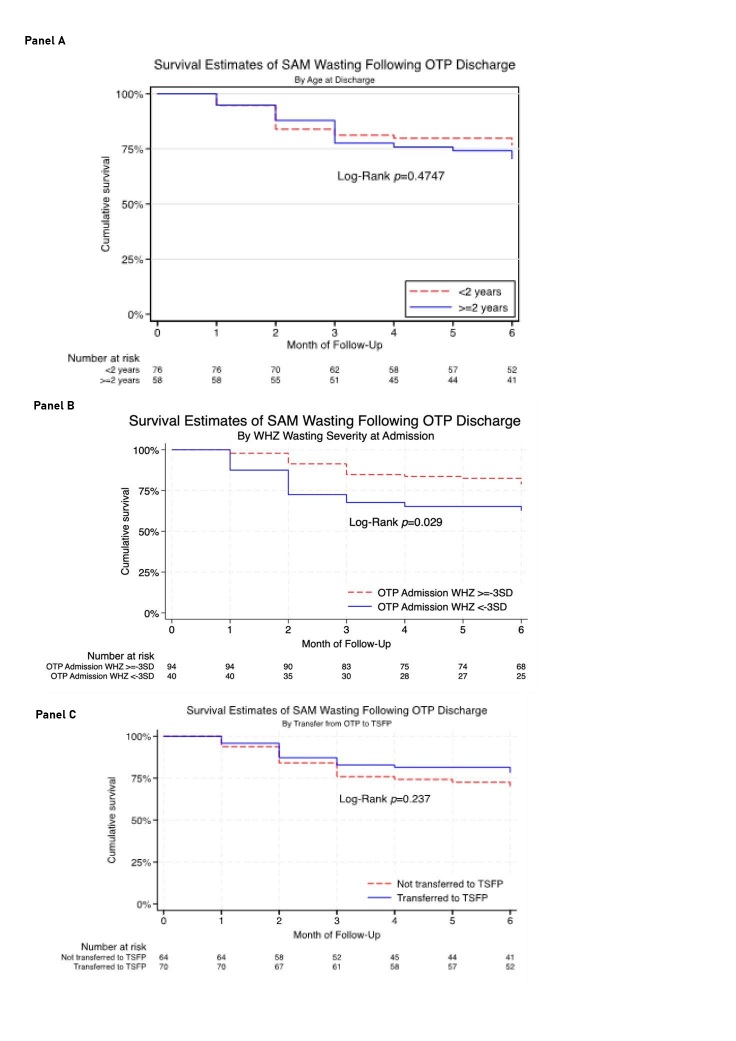
Survival estimates of SAM wasting following OTP discharge **Panel A.** By age. **Panel B.** By wasting severity at admission. **Panel C.** By whether the child was transferred from OTP to TSFP. OTP – outpatient therapeutic programmes, SAM – severe acute malnutrition, TSFP – targeted supplementary feeding programme.

**Table 2** shows the relapse incidence by the analysis cohorts. For SAM relapse using WHZ, the highest incidence of relapse was in the first 3–4 months with 5.2, 9.1, and 6.1% relapsing to SAM at T1, T2 and T3 respectively. From T4 to T6, relapse incidence rate was lower at 1.5, 0.8, and 3.3% respectively. The cumulative incidence rate at T6 was 26.0% (95% CI = 19.3, 34.5%). Rural children had a higher relapse rate (31.4%; 95% CI = 20.5, 46.0%) compared to urban children at T6 (22.7%; 95% CI = 14.9, 33.7%), however this difference was not statistically significant (*P* = 0.285). Hiran region had a higher relapse rate (30.2%) than Bay region (21.8%) at T6, but this difference was also not statistically significant (*P* = 0.309). As shown in **Figure 3**, Panel A, older children (≥ 2 years) showed a slightly higher relapse rate (29.5%) than younger children (23.3%), but not statistically significant (*P* = 0.488). Notably, in **Figure 3**, Panel B, children who had WHZ < −3 SD at admission had significantly higher relapse rates (37.4%) than children admission WHZ ≥ −3 SD (21.2%; *P* = 0.029). As shown in **Figure 3**, Panel C, children who were not transferred from OTP to TSFP had a higher relapse rate at T6 (30.9% *vs*. 21.7% for those referred to TSFP (*P* = 0.237).

**Table 2:** Incidence of wasting for SAM (by WHZ), SAM (by MUAC) and MAM (by WHZ) at each follow-up point

	Overall		Region					Rural urban				
**Follow-up time**	**Incidence, %**	**Cumulative incidence, % (95% CI)**	**Incidence, %**	**Cumulative incidence, % (95% CI)**	**Incidence, %**	**Cumulative incidence, % (95% CI)**	***P*-value**	**Incidence, %**	**Cumulative incidence, % (95% CI)**	**Incidence, %**	**Cumulative incidence, % (95% CI)**	***P*-value**
**SAM by WHZ**												
	(n = 134)		Bay (IDP population) n = 71		Hiran (non-IDP population) n = 63			Rural (n = 51)		Urban (n = 83)		
T1	5.2	5.2 (2.5, 10.6)	4.2	4.2 (1.4, 12.5)	6.3	6.3 (2.4, 16.0)		5.9	5.9 (1.9, 17.1)	4.8	4.8 (1.8, 12.3)	
T2	9.1	14.3 (9.4, 21.5)	10.2	14.4 (8.0, 25.1)	8.0	14.3 (7.7, 25.7)		7.8	13.7 (6.8, 26.7)	9.9	14.7 (8.6, 24.5)	
T3	6.1	20.4 (14.5, 28.3)	4.3	18.7 (11.3, 30.1)	7.9	22.2 (13.8, 34.6)		9.8	23.5 (14.1, 37.7)	3.7	18.4 (11.5, 28.7)	
T4	1.5	21.9 (15.8, 30.0)	0.0	18.7 (11.3, 30.1)	3.2	25.4 (16.4, 38.1)		4.0	27.5 (17.3, 41.9)	0.0	18.4 (11.5, 28.7)	
T5	0.8	22.7 (16.5, 30.9)	0.0	18.7 (11.3, 30.1)	1.6	27.0 (17.7, 39.8)		1.9	29.4 (18.9, 44.0)	0.0	18.4 (11.5, 28.7)	
T6	3.3	26.0 (19.3, 34.5)	3.4	22.1 (13.9, 34.1)	3.2	30.2 (20.5, 43.2)	0.309	2.0	31.4 (20.5, 46.0)	4.3	22.7 (14.9, 33.7)	0.285
**SAM by MUAC**												
	(n = 160)		Bay (IDP population) n = 71		Hiran (non-IDP population) n = 63			Rural (n = 73)		Urban (n = 87)		
T1	8.8	8.8 (5.3, 14.3)	14.9	14.9 (8.5, 25.2)	3.5	3.5 (1.1, 10.4)		2.7	2.7 (0.7, 10.5)	13.8	13.8 (8.1, 23.0)	
T2	2.5	11.3 (7.3, 17.3)	4.1	19.0 (11.7, 29.9)	1.2	4.7 (1.8, 11.9)		0.0	2.7 (0.7, 10.5)	4.7	18.5 (11.7, 28.3)	
T3	1.2	12.5 (8.3, 18.8)	2.8	21.8 (14.0, 33.1)	0.0	4.7 (1.8, 11.9)		0.0	2.7 (0.7, 10.5)	2.3	20.8 (13.7, 31.0)	
T4	0.7	13.2 (8.8, 19.5)	0.0	21.8 (14.0, 33.1)	0.0	4.7 (1.8, 11.9)		0.0	2.7 (0.7, 10.5)	0.0	20.8 (13.7, 31.0)	
T5	0.0	13.2 (8.8, 19.5)	0.0	21.8 (14.0, 33.1)	1.1	5.8 (2.5, 13.4)		1.4	4.1 (1.3, 12.2)	0.0	20.8 (13.7, 31.0)	
T6	0.0	13.2 (8.8, 19.5)	0.0	21.8 (14.0, 33.1)	0.0	5.8 (2.5, 13.4)	0.003*	0.0	4.1 (1.3, 12.2)	0.0	20.8 (13.7, 31.0)	0.002*
**Both SAM and MAM by WHZ**												
	(n = 108)		Bay (IDP population) n = 71		Hiran (non-IDP population) n = 63			Rural (n = 39)		Urban (n = 69)		
T1	26.9	26.9 (19.5, 36.3)	27.4	27.4 (18.0, 40.3)	26.1	26.1 (15.7, 41.3)		28.2	28.2 (16.7, 45.1)	26.1	26.1 (17.3, 38.2)	
T2	9.3	36.2 (28.0, 46.1)	6.6	34.0 (23.7, 47.3)	13.0	39.1 (26.8, 54.7)		10.3	38.5 (25.3, 55.5)	8.9	35.0 (25.0, 47.5)	
T3	2.9	39.1 (30.6, 49.0)	3.4	37.4 (26.7, 50.7)	2.2	41.3 (28.7, 56.8)		2.5	41.0 (27.5, 58.0)	3.0	38.0 (27.7, 50.6)	
T4	4.9	44.0 (35.2, 54.0)	8.9	46.3 (34.7, 59.7)	0.0	41.3 (28.7, 56.8)		0.0	41.0 (27.5, 58.0)	7.9	45.9 (34.9, 58.6)	
T5	3.9	47.9 (38.9, 57.9)	1.8	48.1 (36.4, 61.5)	6.5	47.8 (34.6, 63.0)		7.7	48.7 (34.5, 65.2)	1.6	47.5 (36.4, 60.1)	
T6	2.2	50.1 (41.0, 60.0)	2.2	50.3 (38.3, 63.7)	2.2	50.0 (36.7, 65.1)	0.991	2.6	51.3 (36.8, 67.5)	1.9	49.4 (38.1, 62.0)	0.860

CI– confidence interval, IDP – internally displaced person, MAM – moderate acute malnutrition, MUAC – mid-upper arm circumference, SAM – severe acute malnutrition, T – time, WHZ – weight-for-height z-score

*Values are statistically significant (*P* < 0.05).

For SAM relapse using MUAC, the cumulative incidence was notably lower, 13.2% (95% CI = 8.8, 19.5%) at T6. By month, incidence was highest within 1–3 time points at 8.8, 2.5, and 1.2% respectively. Stratified analyses of wasting by MUAC are shown in Table S2 in the **Online Supplementary Document** but these should be interpreted with caution given small sample sizes and that MUAC severely underestimates the true burden of wasting and relapse [28].

For combined SAM and MAM relapse by WHZ, the cumulative incidence was 50.1% (95% CI = 41.0, 60.0%) at T6, with substantial early relapse rates. Relapse was highest at T1 and T2 at 26.9, and 9.3% respectively. This then tapered to 2.9, 4.9, and 3.9% and 2.2% from T3 to T6 respectively. Combined SAM and MAM relapse showed minimal variation across rural/urban settings (*P* = 0.860) and regions (*P* = 0.991). For combined SAM and MAM, there was no statistically significant difference by severity of WHZ at admission (*P* = 0.335) or by age group (*P* = 0.812).

### Relapse risk factors by WHZ

We investigated a range of potential predictors of SAM relapse using Cohort 1 (SAM relapse by WHZ), including admission characteristics, maternal factors, and child-specific variables (**Table 3**). Both unadjusted and adjusted analyses were performed, with the adjusted model controlling for child age, sex, livelihood zone, and time to relapse.

**Table 3:** Risk ratios of individual and household factors associated with relapse to SAM after discharge from OTP treatment

	Unadjusted		Adjusted*	
**Relapse factors**	**Risk ratio (95% CI)**	***P*-value**	**Risk ratio (95% CI)**	***P*-value**
Admission factors				
Anthropometric at admission				
*WHZ<−3*	2.05 (1.04, 4.03)	0.029†	2.22 (1.04, 4.72)	0.039†
*MUAC<11.5 cm*	1.25 (0.52, 3.03)	0.603	1.86 (0.63, 5.48)	0.262
*HAZ<−2*	0.98 (0.49, 1.95)	0.946	1.09 (0.51, 2.32)	0.831
Length of stay in OTP, days	1.02 (1.00, 1.03)	0.021†	1.02 (1.00, 1.04)	0.027†
Maternal characteristics				
*Mother age (continuous)*	1.02 (0.95, 1.10)	0.560	1.03 (0.95, 1.12)	0.455
*Formal education*	0.85 (0.16, 4.36)	0.838	0.80 (0.15, 4.19)	0.796
*Mother is head of household*	0.56 (0.12, 2.48)	0.424	0.43 (0.09, 2.03)	0.285
*Mother is decision maker on income*	0.65 (0.13, 3.36)	0.597	0.58 (0.11, 3.06)	0.517
*Mother is decision maker on health care*	0.74 (0.14, 3.82)	0.712	0.76 (0.14, 4.03)	0.750
*Mother weight (continuous)*	0.99 (0.97, 1.02)	0.733	0.99 (0.97, 1.02)	0.727
*Mother MUAC measurement (continuous)*	0.98 (0.90, 1.07)	0.602	0.97 (0.89, 1.06)	0.532
*Maternal wasting, MUAC<23 cm*	1.20 (0.53, 2.72)	0.645	1.25 (0.55, 2.83)	0.600
Child feeding indicators				
*Acceptable FCS*	0.52 (0.10, 2.67)	0.413	0.54 (0.10, 2.95)	0.474
Child vaccination & illness history				
*Ever received a vaccination*	0.87 (0.10, 7.24)	0.897	0.93 (0.09, 9.58)	0.949
*Child was ill two weeks prior to survey*	1.15 (0.56, 2.38)	0.688	1.21 (0.58, 2.51)	0.608
Child had access to care when ill				
*Immediately (in the last two weeks)*	0.65 (0.33, 1.29)	0.201	0.65 (0.33, 1.30)	0.224
*Immediately (in the last six month)*	0.95 (0.19, 4.92)	0.954	1.20 (0.22, 6.54)	0.835
Child was admitted in wasting programme (in the last 12 mo)-other than the last admission	0.63 (0.14, 2.80)	0.528	0.53 (0.10, 2.69)	0.443
Child household and environment				
*Household was affected by a recent flooding*	1.43 (0.58, 3.56)	0.421	2.05 (0.69, 6.07)	0.196
*Received BHA cash assistance*	0.52 (0.26, 1.02)	0.044†	0.44 (0.22, 0.90)	0.025†
*Crowded Household (≥5 people)*	1.49 (0.29, 7.67)	0.625	1.56 (0.30, 8.12)	0.594
*Household with ≥3 CU5*	2.24 (0.50, 10.00)	0.267	2.12 (0.47, 9.54)	0.329
*Uses improved toilet*	1.40 (0.17, 11.64)	0.748	1.66 (0.18, 15.09)	0.653
*Uses improved drinking water source*	1.34 (0.16, 11.13)	0.780	1.29 (0.15, 11.36)	0.816
*Lacked access to sufficient quantity of drinking water in last 30 d (≥2 times)*	1.27 (0.28, 5.66)	0.750	1.32 (0.28, 6.14)	0.724
*Have access to handwashing facility*	0.95 (0.11, 7.90)	0.961	0.76 (0.08, 7.06)	0.810
IMAM programme quality				
*Child was transferred from OTP to TSFP*	0.67 (0.34, 1.33)		0.66 (0.32, 1.37)	0.030

BHA – Bureau for Humanitarian Assistance, CI – confidence interval, CU5 – children under five, FCS – food consumption score, HAZ – height-for-age z-score, IMAM – Integrated Management of Acute Malnutrition, MUAC – mid-upper arm circumference, OTP – Outpatient Therapeutic Feeding Programme, TSFP – Targeted Supplementary Feeding Programme, WHZ – weight-for-height z-score.

*Adjusted for child age, sex, livelihood zone and time to relapse.

†Values are statistically significant (*P* < 0.05).

In the adjusted analysis, three factors emerged as significant predictors of SAM relapse. Children with a WHZ below −3 SD at admission had a statistically significantly higher risk of relapse (adjusted HR = 2.22; 95% CI = 1.04, 4.72, *P* = 0.039). The adjusted HR of 2.22 for WHZ < −3 at admission means children with severe wasting at enrolment were more than twice as likely to relapse compared to those with moderate wasting. Similarly, a longer duration of OTP stay was associated with an increased relapse risk (adjusted HR = 1.02 per day; 95% CI = 1.00, 1.04, *P* = 0.027), meaning each additional day in OTP increased relapse risk by 2%. Cash assistance reduced relapse risk by 56%. Additionally, participation in the BHA-funded cash programme was protective against relapse (adjusted HR = 0.44; 95% CI = 0.22, 0.90, *P* = 0.025). Other factors examined, including maternal characteristics (*e.g.* age, education, decision-making power), child feeding practices, vaccination history, and household environment did not show statistically significant associations with relapse risk in the adjusted analysis. While maternal education, water, sanitation and hygiene (WASH) conditions, and feeding practices showed no significant associations, the direction of effects aligns with established nutrition pathways. These findings may reflect sample size limitations or the overwhelming influence of contextual factors like conflict and food insecurity in this setting. Figure S3 in the **Online Supplementary Document** summarises how predictors, protectors and context influence relapse.

## DISCUSSION

To our knowledge, this is the first study of child wasting relapse rates and determinants in the Bay and Hiran regions of Somalia. We found that incidence of SAM at T6 post-OTP exit was as high as 26.0% (22.1% in Bay and 30.2% in Hiran), and incidence of MAM was 50.1% (50.3% in Bay and 50.0% in Hiran). These rates are higher compared to those found in a recent multi-country study, where a 5% cumulative incidence of SAM or all-cause death and 23% acute malnutrition or all-cause death was reported among post-SAM CU5 in Mogadishu, Somalia at six months when employing a MUAC-only acute malnutrition criterion [10]. Notably, the data for our study was collected between November–April which overlap the raining season (deyr) and dry season (Jilaal), while in the multi-country study, data collection occurred during ‘Gu’, the main raining season between harvest (April–June). In our study, the incidence of SAM was highest in the first three follow-up time points and decreased afterwards; similar findings have been reported elsewhere, where the majority of relapse cases occurred in the first three months of follow-up [29]. On the other hand, in a multi-country study that included Somalia, the rate of relapse remained relatively constant over the six-month period at a relapse incidence rate of one case per 100 child-months [10]. Our results of higher incidence of relapse initially during follow-up could be due to multiple contextual factors, including seasonality and major flooding that occurred early on in the follow-up period and affected livelihoods and food security in Bay and Hiran [30]. Relapse persisted throughout follow-up, indicating that relapse risk in Somalia persists over time and children may be at risk both immediately following discharge and for at least six months afterwards, which aligns with previous literature in Somalia and globally [10]. This finding has policy and programmatic implications for ensuring that children’s nutrition, health, and food security are protected and promoted not only through initial wasting recovery, but also through longer-term investments to prevent relapse.

The findings from this analysis demonstrate that relapse rates in the Bay and Hiran regions of Somalia vary by anthropometric indicator, by region, and by demographic factors. When defined by WHZ, the incidence of SAM relapse was 26% and relapse rate was higher in rural areas (31.4% in rural areas *vs.* 22.7% in urban areas), but not statistically significant (*P* = 0.285). Mid-upper arm circumference (MUAC) relapse rate is lower when compared to WHZ at all follow-up time points. Variable relapse rates according to anthropometric criteria have also been reported elsewhere, with one study finding that using a MUAC<115 mm or nutritional oedema criteria missed over 80% of relapse cases in their sample [10,13,28]. These differences in wasting and relapse burden according to measurement metric underscore the critical importance of using a reliable and accurate measure of wasting, such as WHZ, where possible; MUAC alone may underestimate and misclassify wasting risk – an issue that cannot be understated [28].

In our analysis, WHZ at admission to OTP was statistically significantly associated with risk of relapse among CU5. This finding is consistent with previous results that have also shown anthropometry at admission to be associated with acute malnutrition relapse among CU5 [6,10,12,15]. We also found length of stay in the OTP to be associated with wasting relapse risk in our cohort. This finding is consistent with work done in Mali, where researchers likewise found a longer length of stay to be predictive of relapse, and one hypothesis for this association with relapse outcomes is that children staying longer in OTP may have other complications and poorer health in general, resulting in increased nutritional vulnerability even after OTP discharge [12]. In our analysis, participation in the BHA cash assistance programme was protective against relapse. This finding is consistent with previous work from the Democratic Republic of Congo where receiving cash transfers was associated with decreased rates of relapse [6,31]. Interestingly, other factors such as maternal characteristics (*e.g.* age, education, and decision-making power), child feeding practices, vaccination history, and household environment did not show statistically significant associations with relapse risk in our adjusted analysis. However, additional work done by our group has identified factors such as maternal age, MUAC, and education, household WASH environment, and child vaccination and diet as drivers of wasting in the Bay and Hiran regions [32]. Additionally, previous work in Somalia has shown that living in a household experiencing severe hunger or falling into the lowest wealth quartile were risk factors for relapse in post-SAM children [10]. Other analyses in sub-Saharan Africa have found such factors as child sickness and vaccination, child dietary diversity, maternal/caretaker age, maternal literacy, hygiene practices, and household water source and food insecurity to be associated with acute malnutrition relapse among CU5 [6,12,14,15,23,33,34]. In light of contrasting evidence, differing definitions of relapse, and the known relationship between factors such as food security and nutrition outcomes, our findings on determinants of relapse risk should be interpreted cautiously given the relatively wide confidence intervals for some estimates.

Our findings highlight critical needs across the continuum of SAM caregiven 49% of children who exited OTP were not transferred to TSFP in our study. Treatment services require strengthening, with evidence from Ethiopia showing poor adherence to guidelines and inadequate staffing [35]. Discharge practices need improvement, as demonstrated by premature discharge cases in our study and others [6,10–12,14,33]. Post-discharge monitoring must be enhanced through community-based approaches and early warning systems. While family-MUAC monitoring was implemented in Somalia in 2020 [36], additional approaches like weekly MUAC reporting and regular follow-up are needed [6]. For prevention, while small quantity lipid-based nutrient supplements show promise for preventing child wasting, research on its utility for prevention of wasting relapse is limited [37,38].

The disparity in relapse rates by anthropometric criteria (26% WHZ *vs.* 13.2% MUAC) underscore the need for standardised definitions and measurements of wasting relapse [6,28,39]. This standardisation is crucial for effective resource allocation and treatment planning in humanitarian settings.

Our study was limited by smaller-than-planned recruitment due to access constraints, though the sample size (> 30) was adequate for our analyses [40]. While lacking a non-post-SAM control group, our diverse sample allowed assessment of relapse patterns across different subpopulations. We propose future studies that extend beyond seven months of follow-up, as evidence shows children remain at risk of poor outcomes over longer periods [6,41]. Larger, longer-term studies across multiple regions of Somalia would enable analysis of seasonal trends in relapse [42] and strengthen understanding of relapse determinants. Research priorities include evaluating relapse prevention approaches like small quantity lipid-based nutrient supplements, Blanket Supplementary Feeding Program, Cash and discharge criteria modification [6,37], and developing standardised definitions of relapse to improve measurement consistency and resource utilisation [28,39]. Well-powered studies across diverse populations are needed to better characterise both relapse rates and their underlying causes throughout Somalia.

## CONCLUSIONS

With SAM relapse rates as high as 26% and MAM as high as 50% at seven months among children, it is without a doubt clear that wasting relapse is a serious public health concern in Somalia. Moreover, the burden that relapse places on humanitarian resources by repeatedly treating the same children and the risk that relapse poses to children’s health and survival, suggests that addressing relapse should be top of funding, policy and programming priorities in the country. The key drivers of relapse identified in this study shed some light on ways to intervene however further research is needed. Additionally, the variable rates of relapse according to anthropometric criteria emphasises the need for future work to adopt an accurate and reliable definition of wasting relapse for more effective resource distribution and identification and treatment of relapsed children. Future priorities must include standardising WHZ-based relapse definitions globally and funding transitional support programmes for the critical six-month post-discharge period. Research should focus on integrated cash-nutrition interventions and early warning systems for conflict-affected settings like Somalia.

## Additional material


Online Supplementary Document

## References

[1] United Nations Children’s Fund; World Health Organization; The World Bank. Levels and trends in child malnutrition: UNICEF/WHO/World Bank Group Joint Child Malnutrition Estimates: Key findings of the 2023 edition. New York, USA: United Nations Children’s Fund, World Health Organization; 2023. Available: https://www.who.int/publications/i/item/9789240112308. Accessed: 9 February 2025.

[2] United Nations Children’s Fund. Child Malnutrition. 2023. Available: https://data.unicef.org/topic/nutrition/malnutrition/. Accessed: 9 February 2025.

[3] Akuu JA Amagnya MA Community-based management of acute malnutrition: Implementation quality, and staff and user satisfaction with services. J Taibah Univ Med Sci. 2023;18:988–96. 10.1016/j.jtumed.2023.02.002 36890797 PMC9986645

[4] Schaefer R Mayberry A Briend A Manary M Walker P Stobaugh H Relapse and regression to severe wasting in children under 5 years: A theoretical framework. Matern Child Nutr. 2021;17:e13107. 10.1111/mcn.13107 33145990 PMC7988852

[5] Girma T James PT Abdissa A Luo H Getu Y Fantaye Y Nutrition status and morbidity of Ethiopian children after recovery from severe acute malnutrition: Prospective matched cohort study. PLoS One. 2022;17:e0264719. 10.1371/journal.pone.0264719 35271590 PMC8912152

[6] Stobaugh HC Mayberry A McGrath M Bahwere P Zagre N Manary M Relapse after severe acute malnutrition: A systematic literature review and secondary data analysis. Matern Child Nutr. 2019;15:e12702. 10.1111/mcn.12702 30246929 PMC6587999

[7] Childhood Acute Illness and Nutrition (CHAIN) Network Childhood mortality during and after acute illness in Africa and south Asia: a prospective cohort study. Lancet Glob Health. 2022;10:e673–84. 10.1016/S2214-109X(22)00118-8 35427524 PMC9023747

[8] World Bank. Somalia At-A-Glance. 2023. Available: https://www.worldbank.org/en/country/somalia. Accessed: 9 February 2025.

[9] King S D’Mello-Guyett L Yakowenko E Riems B Gallandat K Mama Chabi S A multi-country, prospective cohort study to measure rate and risk of relapse among children recovered from severe acute malnutrition in Mali, Somalia, and South Sudan: a study protocol. BMC Nutr. 2022;8:90. 10.1186/s40795-022-00576-x 36002905 PMC9404649

[10] King S Marshak A D’Mello-Guyett L Yakowenko E Chabi SM Samake S Rates and risks factors for relapse among children recovered from severe acute malnutrition in Mali, South Sudan, and Somalia: a prospective cohort study. Lancet Glob Health. 2025;13:e98–e111. 10.1016/S2214-109X(24)00415-7 39706667

[11] Lambebo A Temiru D Belachew T Frequency of relapse for severe acute malnutrition and associated factors among under five children admitted to health facilities in Hadiya Zone, South Ethiopia. PLoS One. 2021;16:e0249232. 10.1371/journal.pone.0249232 33765081 PMC7993841

[12] Kangas ST Coulibaly IN Tausanovitch Z Ouologuem B Marron B Radin E Post-Recovery Relapse of Children Treated with a Simplified, Combined Nutrition Treatment Protocol in Mali: A Prospective Cohort Study. Nutrients. 2023;15:2636. 10.3390/nu15112636 37299599 PMC10255596

[13] Guesdon B Katwal M Poudyal AK Bhandari TR Counil E Nepali S Anthropometry at discharge and risk of relapse in children treated for severe acute malnutrition: a prospective cohort study in rural Nepal. Nutr J. 2021;20:32–7. 10.1186/s12937-021-00684-7 33820545 PMC8021301

[14] Bliznashka L Grantz KH Botton J Berthé F Garba S Hanson KE Burden and risk factors for relapse following successful treatment of uncomplicated severe acute malnutrition in young children: Secondary analysis from a randomised trial in Niger. Matern Child Nutr. 2022;18:e13400. 10.1111/mcn.13400 35866201 PMC9480908

[15] Adegoke O Arif S Bahwere P Harb J Hug J Jasper P Incidence of severe acute malnutrition after treatment: A prospective matched cohort study in Sokoto, Nigeria. Matern Child Nutr. 2021;17:e13070. 10.1111/mcn.13070 32761792 PMC7729648

[16] The Fund For Peace. Fragile States Index. 2023. Available: https://fragilestatesindex.org/global-data/. Accessed: 9 February 2025.

[17] Somalia National Bureau of Statistics. Somalia Poverty Report. 2023. Available: https://nbs.gov.so/wp-content/uploads/2024/08/Somalia-Poverty-Report-2023.pdf. Accessed: 9 February 2025.

[18] Integrated Food Security Phase Classification. Somalia: Acute Food Insecurity Situation for January–March 2024 and Projection for April–June 2024. 2024. Available: https://www.ipcinfo.org/ipc-country-analysis/details-map/en/c/1156834/. Accessed: 9 February 2025.

[19] Food Security and Nutrition Analysis Unit - Somalia. Livelihood Baseline Analysis Bay and Bakool. Technical Series Report No VI. 23. 2009. Available: https://fsnau.org/downloads/Bay-Bakool-Rural-Baseline-Analysis-Report.pdf. Accessed: 27 September 2024.

[20] IGAD Climate Prediction & Applications Centre. Climate Trends over Hiiraan Region, Hirshabelle. 2020. Available: https://www.icpac.net/documents/315/Policy_Brief_Hiraan_Region_Hirshabelle.pdf. Accessed: 27 September 2024.

[21] European Union Agency for Asylum. Country Guidance: Somalia. Common analysis and guidance note. 2nd edition. Luxembourg: Publications Office of the European Union; 2023. Available: https://www.euaa.europa.eu/sites/default/files/publications/2023-08/2023_Country_Guidance_Somalia.pdf. Accessed: 27 September 2024.

[22] Somali Federal Republic Ministry of Health and Human Service. Somali Guidelines for Integrated Management of Acute Malnutrition. Mogadishu, Somalia: Government of Somalia; 2021. Available: https://reliefweb.int/report/somalia/somali-guidelines-integrated-management-acute-malnutrition. Accessed: 27 September 2024.

[23] Abitew DB Yalew AW Bezabih AM Bazzano AN Predictors of relapse of acute malnutrition following exit from community-based management program in Amhara region, Northwest Ethiopia: An unmatched case-control study. PLoS One. 2020;15:e0231524. 10.1371/journal.pone.0231524 32320426 PMC7176369

[24] Walton S, Alier KK, Garretson S, Grounds S, Khattak Q, Tripaldi M, et al. A Cluster-Randomized Trial to Compare Effectiveness and Cost-effectiveness of Cash Plus Interventions in Preventing Child Wasting in Somalia: An Evidence-Based Methodology. medRxiv: 25322679 [preprint]. 2025. Available: https://www.medrxiv.org/content/10.1101/2025.02.21.25322679v1. Accessed: 10 February 2025. 10.1101/2025.02.21.25322679

[25] WHO Multicentre Growth Reference Study Group WHO Child Growth Standards based on length/height, weight and age. Acta Paediatr Suppl. 2006;450:76–85. 16817681 10.1111/j.1651-2227.2006.tb02378.x

[26] Leroy JL. Zscore06: Stata command for the calculation of anthropometric z-scores using the 2006 WHO child growth standards. 2011. Available: http://www.ifpri.org/staffprofile/jef-leroy. Accessed: 10 February 2025.

[27] STROBE. Strengthening the Reporting of Observational Studies in Epidemiology. Available: https://www.strobe-statement.org/. Accessed: 10 February 2025.

[28] Garretson S Walton S Alier KK Grounds S Khattak Q Mohamoud SA Analysing concordance between MUAC, MUACZ, and WHZ in diagnosing acute malnutrition among children under five in Somalia. J Glob Health. 2025;15:04258. 10.7189/jogh.15.04258 41383165 PMC12699285

[29] Dale NM Salim L Lenters L Sadruddin S Myatt M Zlotkin SH Recovery and relapse from severe acute malnutrition after treatment: a prospective, observational cohort trial in Pakistan. Public Health Nutr. 2018;21:2193–9. 10.1017/S1368980018000745 29615143 PMC11106019

[30] UNOCHA. Somalia: 2023 Deyr Season Floods Weekly Situation Report No. 4. 2023. Available: https://www.unocha.org/publications/report/somalia/somalia-2023-deyr-season-floods-situation-report-no-4-10-december-2023. Accessed: 10 February 2025.

[31] Grellety E Babakazo P Bangana A Mwamba G Lezama I Zagre NM Effects of unconditional cash transfers on the outcome of treatment for severe acute malnutrition (SAM): a cluster-randomised trial in the Democratic Republic of the Congo. BMC Med. 2017;15:87. 10.1186/s12916-017-0848-y 28441944 PMC5405483

[32] Grounds S, Walton S, Alier KK, Garretson S, Mohamoud SA, Abdikadir S, et al. Assessing the Drivers of Wasting among Children Under 5 and Their Mothers in The Bay and Hiran Regions of Somalia. medRxiv: 25322675 [preprint]. 2025. Available: https://www.medrxiv.org/content/10.1101/2025.02.21.25322675v1. Accessed: 10 February 2025. 10.1101/2025.02.21.25322675

[33] Somassè YE Dramaix M Bahwere P Donnen P Relapses from acute malnutrition and related factors in a community-based management programme in Burkina Faso. Matern Child Nutr. 2016;12:908–17. 10.1111/mcn.12197 26059267 PMC6860074

[34] Abitew DB Worku A Mulugeta A Bazzano AN Rural children remain more at risk of acute malnutrition following exit from community-based management of acute malnutrition program in South Gondar Zone, Amhara Region, Ethiopia: a comparative cross-sectional study. PeerJ. 2020;8:e8419. 10.7717/peerj.8419 32071802 PMC7008819

[35] Alelign D Fentahun N Yigzaw ZA Barriers and facilitators of severe acute malnutrition management at Felege Hiwot Comprehensive Specialized Hospital, Bahir Dar, North West Ethiopia, descriptive phenomenological study. PLoS One. 2024;19:e0299575. 10.1371/journal.pone.0299575 38512842 PMC10956781

[36] Omar M. Family led measurement of the Mid-Upper Arm Circumference (MUAC). United Nations Children’s Fund. 2024. Available: https://www.unicef.org/somalia/stories/family-led-measurement-muac. Accessed: 27 September 2024.

[37] Airbel Impact Lab. SQ-LNS to reduce relapse post-discharge from acute malnutrition treatment. Available: https://airbel.rescue.org/projects/sq-lns-to-reduce-relapse-post-discharge-from-acute-malnutrition-treatment/. Accessed: 27 September 2024.

[38] United Nations Children’s Fund. Small Supplements for the Prevention of Malnutrition in Early Childhood (Small Quantity Lipid-based Nutrient Supplements), Brief Guidance Note. New York, USA: United Nations Children’s Fund. 2023. Available: https://www.unicef.org/media/134786/file/SQLNS_Brief_Guidance_Note.pdf. Accessed: 27 September 2024.

[39] O’Sullivan NP Lelijveld N Rutishauser-Perera A Kerac M James P Follow-up between 6 and 24 months after discharge from treatment for severe acute malnutrition in children aged 6-59 months: A systematic review. PLoS One. 2018;13:e0202053. 10.1371/journal.pone.0202053 30161151 PMC6116928

[40] Boston University School of Public Health. LaMorte W. Central Limit Theorem. Available: https://sphweb.bumc.bu.edu/otlt/MPH-Modules/BS/BS704_Probability/BS704_Probability12.html. Accessed: 27 September 2024.

[41] Chang CY Trehan I Wang RJ Thakwalakwa C Maleta K Deitchler M Children Successfully Treated for Moderate Acute Malnutrition Remain at Risk for Malnutrition and Death in the Subsequent Year after Recovery. J Nutr. 2013;143:215–20. 10.3945/jn.112.168047 23256140 PMC3735907

[42] Luc G, Keita M, Bazongo B, Diarra B, Virchenko A, Okley C, et al. Special focus: Wasting patterns in Somalia. Emergency Nutrition Network. 2024. Available: https://www.ennonline.net/fex/72/special-focus-wasting-patterns-somalia. Accessed: 27 September 2024.

